# Triage Accuracy and the Safety of User-Initiated Symptom Assessment With an Electronic Symptom Checker in a Real-Life Setting: Instrument Validation Study

**DOI:** 10.2196/55099

**Published:** 2024-09-26

**Authors:** Ville Liu, Minna Kaila, Tuomas Koskela

**Affiliations:** 1 Faculty of Medicine University of Helsinki Helsinki Finland; 2 Public Health Medicine Faculty of Medicine University of Helsinki Helsinki Finland; 3 Department of General Practice Faculty of Medicine and Health Technology Tampere University Tampere Finland; 4 The Wellbeing Services County of Pirkanmaa Tampere Finland

**Keywords:** nurse triage, emergency department triage, triage, symptom assessment, health services accessibility, telemedicine, eHealth, remote consultation, eHealth, primary health care, primary care, urgent care, health services research, health services

## Abstract

**Background:**

Previous studies have evaluated the accuracy of the diagnostics of electronic symptom checkers (ESCs) and triage using clinical case vignettes. National Omaolo digital services (Omaolo) in Finland consist of an ESC for various symptoms. Omaolo is a medical device with a Conformité Européenne marking (risk class: IIa), based on Duodecim Clinical Decision Support, EBMEDS.

**Objective:**

This study investigates how well triage performed by the ESC nurse triage within the chief symptom list available in Omaolo (anal region symptoms, cough, diarrhea, discharge from the eye or watery or reddish eye, headache, heartburn, knee symptom or injury, lower back pain or injury, oral health, painful or blocked ear, respiratory tract infection, sexually transmitted disease, shoulder pain or stiffness or injury, sore throat or throat symptom, and urinary tract infection). In addition, the accuracy, specificity, sensitivity, and safety of the Omaolo ESC were assessed.

**Methods:**

This is a clinical validation study in a real-life setting performed at multiple primary health care (PHC) centers across Finland. The included units were of the walk-in model of primary care, where no previous phone call or contact was required. Upon arriving at the PHC center, users (patients) answered the ESC questions and received a triage recommendation; a nurse then assessed their triage. Findings on 877 patients were analyzed by matching the ESC recommendations with triage by the triage nurse.

**Results:**

Safe assessments by the ESC accounted for 97.6% (856/877; 95% CI 95.6%-98.0%) of all assessments made. The mean of the exact match for all symptom assessments was 53.7% (471/877; 95% CI 49.2%-55.9%). The mean value of the exact match or overly conservative but suitable for all (ESC’s assessment was 1 triage level higher than the nurse’s triage) symptom assessments was 66.6% (584/877; 95% CI 63.4%-69.7%). When the nurse concluded that urgent treatment was needed, the ESC’s exactly matched accuracy was 70.9% (244/344; 95% CI 65.8%-75.7%). Sensitivity for the Omaolo ESC was 62.6% and specificity of 69.2%. A total of 21 critical assessments were identified for further analysis: there was no indication of compromised patient safety.

**Conclusions:**

The primary objectives of this study were to evaluate the safety and to explore the accuracy, specificity, and sensitivity of the Omaolo ESC. The results indicate that the ESC is safe in a real-life setting when appraised with assessments conducted by triage nurses. Furthermore, the Omaolo ESC exhibits the potential to guide patients to appropriate triage destinations effectively, helping them to receive timely and suitable care.

**International Registered Report Identifier (IRRID):**

RR2-10.2196/41423

## Introduction

### Background

Seeking information online regarding medical symptoms is a common and well-known phenomenon among people and patients worldwide [1-4]. Usually, the general public searches online for symptoms associated with their medical condition before receiving a medical diagnosis. This includes websites of support groups, written blogs by patients, websites created by editors of popular media, governmental sites, and artificial intelligence (AI) interfaces. However, self-diagnostic web-based sources may be of varying quality in terms of reliability, with misleading information and possibly false advertising [5,6].

To address these problems, digital health care applications are spreading online, including self-diagnosis tools and electronic symptom checkers (ESCs) [7-10]. These are meant to provide solutions and information to the user seeking to learn more about symptoms or a condition they have or think they might have. In cases where access to human health care experts may be limited, telehealth services have tremendous promise for transforming the provision of health care services [11]. Conversely, studies find that healthier users use digital services more often than others and are also more likely to be younger, female, and more highly educated, and to have higher income levels [10,12,13].

Based on the user’s input, ESCs use algorithms to make diagnostic suggestions, offer advice on what action to take, and help in identifying the relevant condition. This is medical triage, and it involves directing patients to the most suitable location within an appropriate time frame. In clinical practice, triage assessment and guidance are usually done by health care professionals either over the phone or face to face, for example, at a health care center [14]. Triage takes up a lot of professionals’ time and its quality varies. Therefore, even the partial digitalization of triage in health care organizations could increase service uniformity, enhance efficiency, and free up working hours [15,16]. This inherently requires that health organizations and teams reorganize their workflows and work distributions to support clinical processes [17,18].

The Omaolo ESC questionnaires and algorithms in use are based on research evidence, probabilities, and expert opinions as to whether the condition described is mild and self-limiting. In terms of urgency, an assessment is made on how soon the condition would worsen without treatment or whether it requires the intervention of a health care professional. However, as with clinical decision-making in general, making an accurate diagnosis requires user-provided information, clinical examinations, various diagnostic tests, and potential consultations with other health care professionals [19,20].

Previous studies have evaluated the accuracy of the diagnostics of ESCs and triage using clinical case vignettes [21-27]. Variation exists between different ESCs, and the conditions being assessed, including the triage capabilities [8,27-29]. In some studies, the diagnostic accuracy of clinicians has been shown to be superior in both primary and specialized health care when compared with ESC tools [22,23]. These studies have shown that users may be referred to as self-care even if they need professional help, and users for whom self-care would suffice are referred to unnecessary counseling. There are risks and the potential for error in ESC-based triage [8,21-29]. In particular, self-care guidance should be limited to cases where it is safe and appropriate. There is currently a limited amount of evidence available on the impact of ESCs on seeking treatment with real-life users [12,30]. However, respondents were satisfied with the ESC services they use [13,31,32].

A study comparing the accuracy of physicians’ and computer diagnostics found that physicians listed the correct diagnosis first more often across all study vignettes compared with ESCs (79.1%-65.3% vs 40.5%-24.3%; *P* <.001) as well as in the top 3 diagnoses listed (84.3% vs 51.2%; *P* <.001) [24]. There is limited evidence of live clinical patient safety hazards associated with the use of ESCs, as safety has mainly been evaluated with the use of clinical vignettes [7,8,21,23,24,26]. When comparing AI and human doctors concerning triage and diagnosis, some AI systems were able to provide triage and diagnostic information on a level of clinical accuracy and safety comparable to human doctors [8,23,33]. However, ESCs on average make the user’s triage more sensitive to the need for more urgent care than the user would need [22,34].

The seamless integration of ESCs into the broader health care triage process is crucial for achieving their intended goals, such as preventing emergency departments overcrowding and providing more accurate symptom assessment and triage for citizens. ESCs can offer citizens a preliminary triage level for their symptoms before contacting health care services [35]. In addition, ESCs and eHealth applications can serve an educational purpose by providing users with structured, research-based disease and treatment information that is easily accessible [36,37]. From a clinical perspective, the ability to accurately identify cases where self-care suffices is paramount in assessing an app’s utility in preventing overcrowding and the “unnecessary use of healthcare services” [38].

### Description of the Omaolo Electronic Symptom Checkers

Omaolo is a national web-based service for health care and social welfare. The purpose of Omaolo is to promote the health and well-being of citizens. Omaolo supports self-care and helps people to contact public health care professionals, if necessary. Omaolo is a medical device with a Conformité Européenne marking, manufactured by government-owned DigiFinland and Duodecim Publishing Company Ltd. Omaolo was granted a CE certificate in accordance with the requirements of European Union Regulation 2017/745 (Medical Device Regulation) in May 2022 [39]. The aim of the Omaolo ESC is to identify, based on an assessment of alarm symptoms and other pre-existing conditions, situations that require immediate or urgent assessment and to conduct follow-up examinations and treatment without delay in situations where conservative treatment may lead to complications. The questionnaires and the algorithms the ESC uses are based on evidence and legal requirements [40].

The ESC operates as [41] the user initially receives reliable information about the symptom (articles from the Health Library Duodecim) and a short summary. If unable to decide on the course of action needed, the user can answer the ESC’s questions. The ESC will then suggest the estimated needed treatment and its urgency. The results of the completed survey made by identified users are saved and prompted to be sent to a regional health care professional through Omaolo. The ESC algorithm initially seeks to identify alarming symptoms and then prompt the user to contact the nearest emergency department immediately. The idea is to identify situations where a professional assessment is necessary and to determine the urgency of the assessment. The user is encouraged to consider whether they may have symptoms that are not covered by the information or survey provided. As the questionnaires might not cover all possible situations that could be due to other illnesses, treatments, or other causes that the user may have, the following help text is displayed to the user at the end of the query: “If you have symptoms that have not been covered in the survey or other illnesses or medications that you think affect your need for treatment, contact your PHC provider or, in an emergency, the nearest emergency department.” [41]. Description of the Omaolo service is described in detail in the study protocol [40].

### Objectives

We hypothesize that the Omaolo ESC assessments are safe to use compared with the assessment of a triage nurse.

The aim is to study the clinical validity of the Omaolo ESC and to evaluate its exact triage accuracy, specificity, sensitivity, and safety. These parameters can be used to determine if Omaolo ESC can direct the right patient user to the right place at the right time. The main research question was: how well does triage by the ESC match the triage of a nurse [40] (Multimedia Appendix 1)?

## Methods

### Study Design and Setting

The study setting was primary health care (PHC), and the data were collected at 18 PHC centers nationwide in Finland. The data were collected between June 1, 2018, and December 31, 2020. The study used the version of the Omaolo ESC that was in use in 2018. Each participating organization provided a study space where it is practically possible for the users (patients) to complete the ESC questionnaire and a nurse with at least 2 years’ experience of triage nurse work in primary care to perform triage (Textbox 1).

**Nurse’s form questions.**

- How did the user arrive at the reception (walk-in or via telephone contact)?
- Did you consult a doctor to assess the user’s need for treatment (triage)?
- What was the most significant thing (observation, symptom, or discovery) that influenced your decision-making?
- Do you feel the need to change your assessment of the need for treatment (triage) after seeing the responses and recommendations of the electronic symptom checker? (yes or no)
- If yes, why did you change the assessment of the need for treatment (triage) after seeing the responses and recommendation of the electronic symptom checker?
- If you feel it is necessary to change your triage assessment, reselect where the user should be referred to according to the classification terms of the electronic symptom checker recommendation.

A total of 119 individual nurses took part in this study. A nurse’s average age was 40 (SD 10.5) years (median 37, IQR 33-47 years), and their average amount of work experience in triage was 9.9 (SD 8.5) years (median 7, IQR 4-14 years).

In total, 3 in every 10 recruited patient users arrived through walk-in at the PHC centers, and the rest first contacted their center through telephone. Upon physically arriving at the PHC centers, the patients were asked if they were willing to participate in the study.

**Figure 1:**
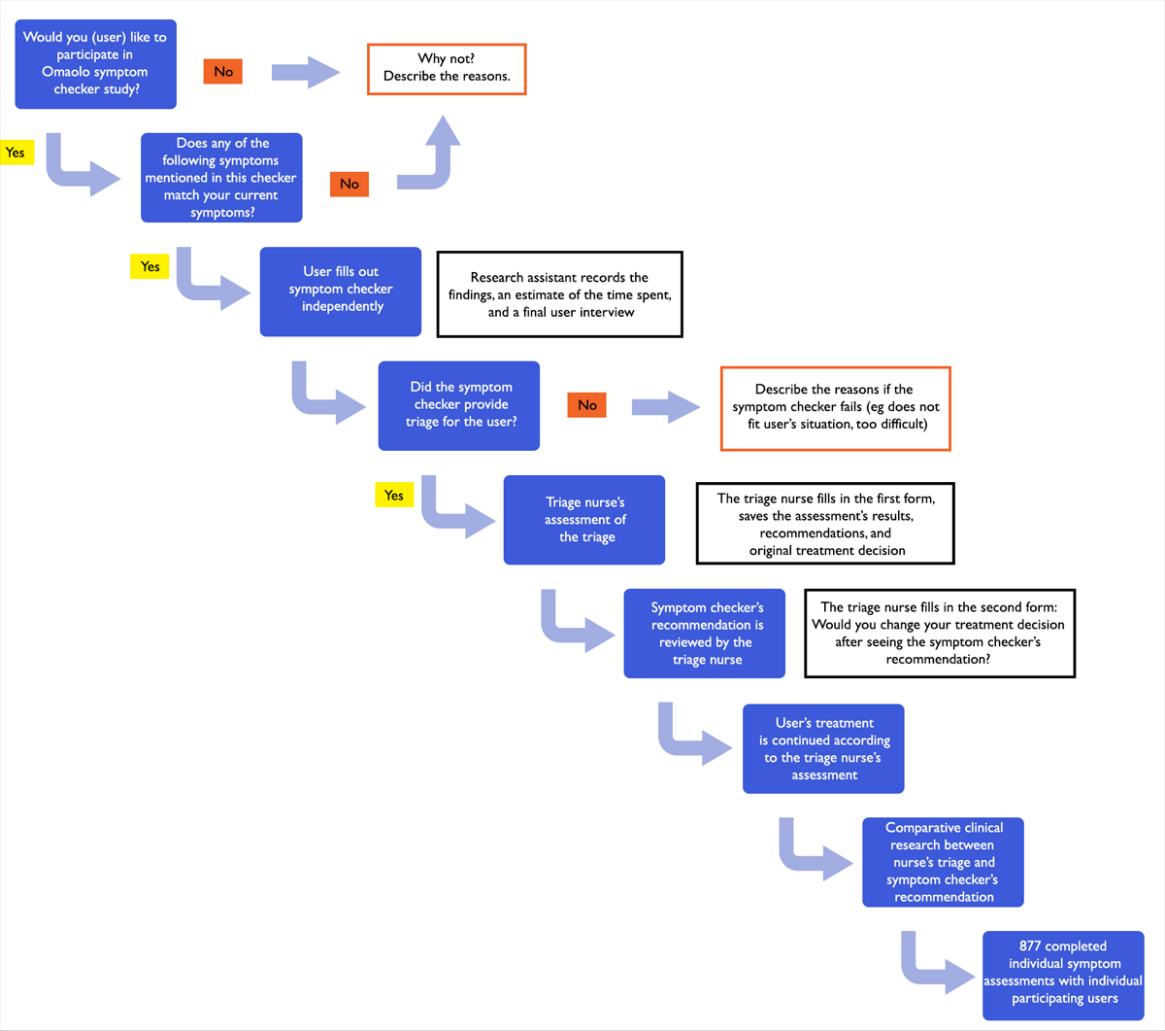
Flowchart showing the collection of the symptom checker and nurse triage data.

The patients answered the ESC when they arrived at the PHC center (on arrival at the center, they also filled out a consent form). Filling out the questions of the ESC was done in a separate quiet space without the research assistant interfering. Next, a triage nurse made a triage assessment of the same patient and filled out the study questionnaire related to the assessment of the patient’s triage. The triage nurses did not get to know the result of the ESC until they had assessed the patient’s condition themselves. After completing their questionnaire, the triage nurses finally got to see the results of the ESC triage concerning the same patient. Based on that result, the triage nurses filled out another questionnaire inquiring whether they felt it necessary to change their own assessment-based action recommendation after seeing the action recommendation of the ESC. The organization also ensured that the patient population of the study remained unscreened. No user-identifying age or gender data were collected for this study [40] (Figure 1).

### Assessment of Electronic Symptom Checker Coverage and Triage Suggestion Levels

The results of the ESC triage and the assessments of nurses were analyzed from the completed study forms. Each assessment was first analyzed individually, and the results concerning particular symptom assessments were combined.

**Figure 2:**
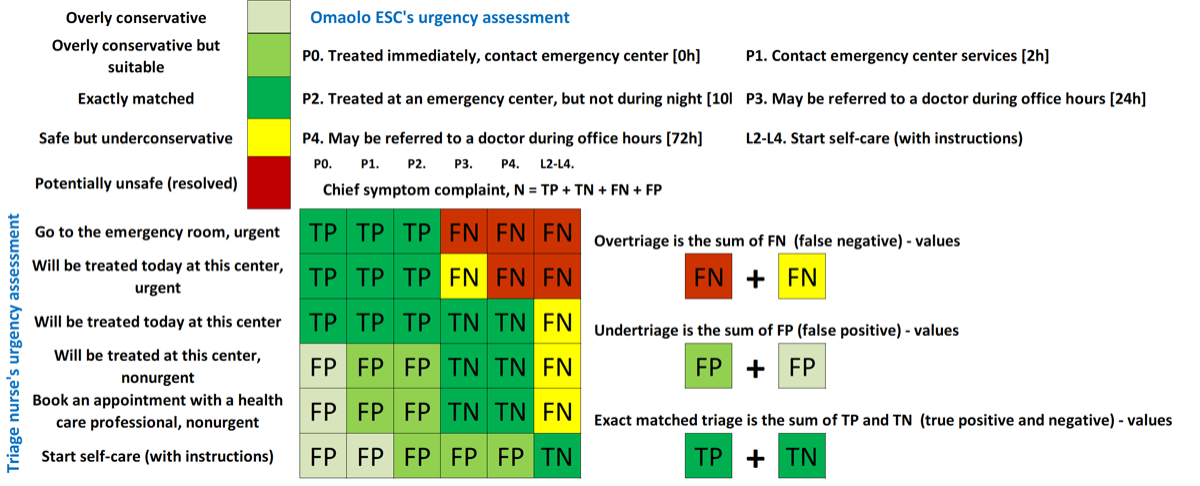
An example showing how the triage comparison chart (confusion matrices) in Figure 3 was constructed with color-coded differences in triage levels (overly conservative, overly conservative but suitable, exactly matched, safe but under conservative, and potentially unsafe [resolved]). Matching rows (triage nurse) to their respective columns (symptom assessment) results in a safety assessment. P0-P4=classification of emergency care criteria. L2-4=referral urgency classes. The columns show how often, by ESC, the symptom assessment was overestimated, underestimated, or accurately matched compared with the assessment made by the triage nurse and the decision made on further referral. ESC: electronic symptom checkers; FN: false negative; FP: false positive; TN true negative; TP: true positive.

**Figure 3:**
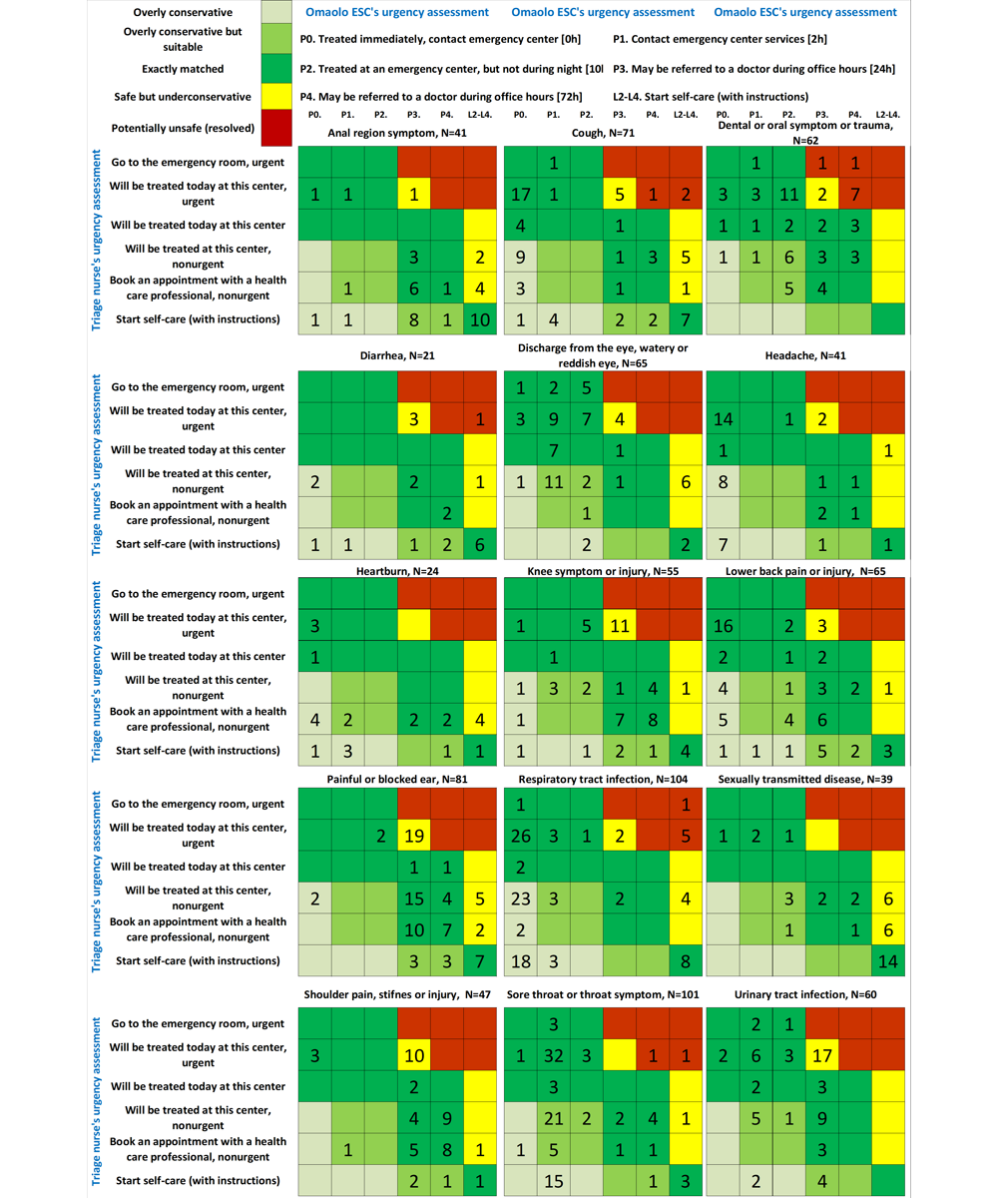
Triage comparison chart (confusion matrices) with color-coded differences in triage levels. ESC: electronic symptom checker.

**Figure 4:**
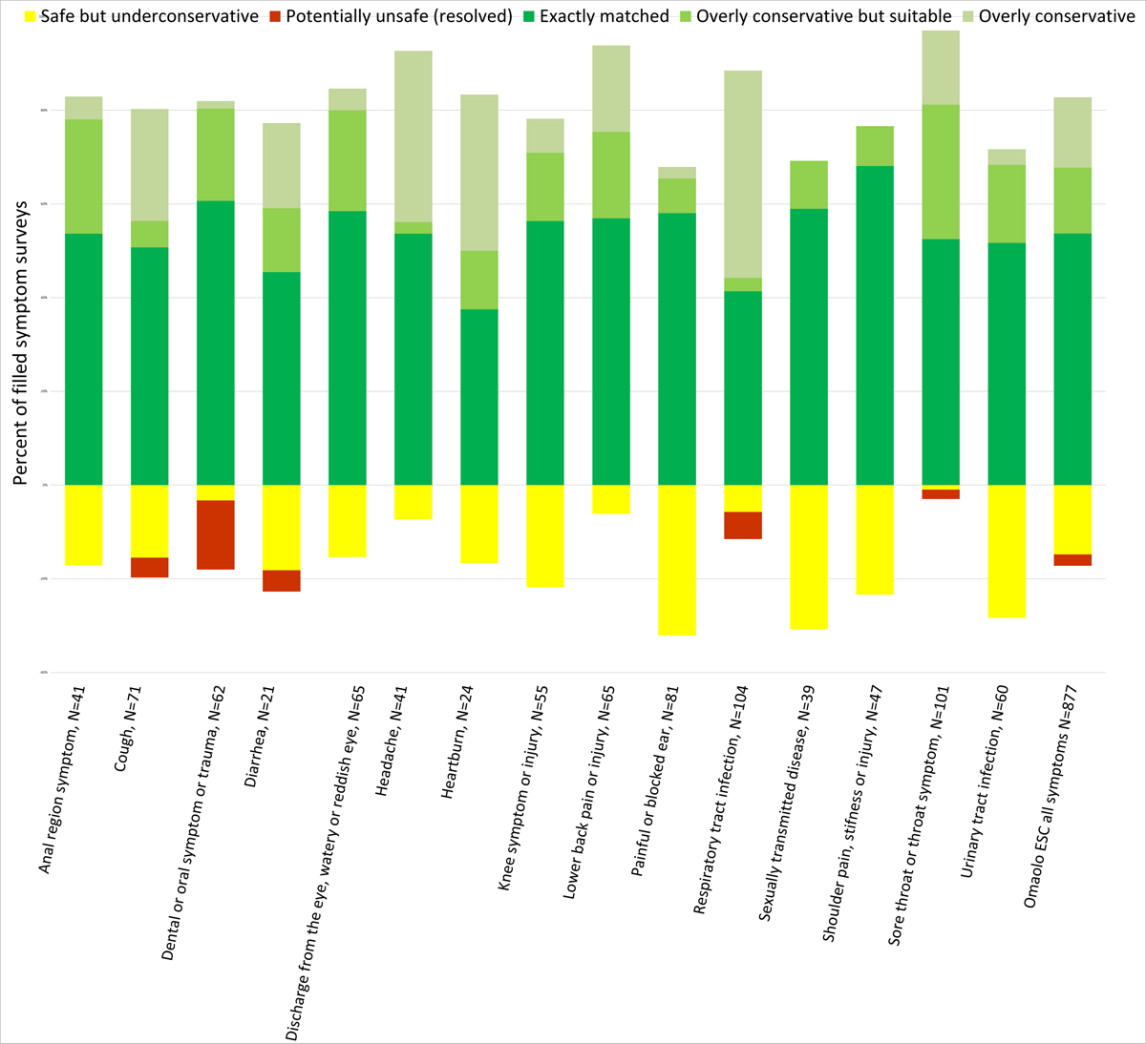
The individual bar heights (y-axis, %) reflect the proportional correspondence and accuracy levels of the electronic symptom checker triage with the nurse’s triage (overly conservative, overly conservative but suitable, exactly matched, safe but under conservative, and potentially unsafe [resolved]). Individual electronic symptom checkers are depicted on the x-axis.

The Ministry of Social Affairs and Health (MSAH) has provided a practical classification for levels of emergency and the Finnish Institute for Health and Welfare (THL) referral class classification with codes [42] (Figures 2-4).

These classification levels are as follows ranging from P0 to P4 and L2 to L4:

- P0: Treated immediately, contact emergency center (within 0 h)
- P1: Contact emergency center services (within 2 h)
- P2: Treated at an emergency center, but not during the night (within 10 h)
- P3: May be referred to a doctor during office hours (within 24 h)
- P4: May be referred to a doctor during office hours (within 72 h)
- L2-L4: Start self-care (with instructions)

Making use of the MSAH emergency levels, the Omaolo ESC and nurse triage were matched as, (1) exactly matched (ESC’s and triage nurse’s triage were the same), (2) overly conservative but suitable (ESC’s assessment was 1 triage level higher than the nurse’s triage), (3) safe but under conservative (ESC’s triage level was 1 triage level lower than the nurse’s triage), (4) overly conservative (ESC’s triage level was 2 levels higher than the nurse’s triage), and (5) potentially unsafe (triage nurse assessed as urgent or an on-call duty but ESC assessed as nonurgent or self-care).

### Assigning Accuracy Gold Standards to the Triage Nurse and the Electronic Symptom Checker

The nurse triage assessment is the gold standard, to which the recommendations of the ESC were compared. An assessment was also defined as potentially unsafe if a case was assessed by the nurse as urgent or an on-call duty but assessed by the ESC as nonurgent or self-care. These assessments were further investigated to ensure patient safety.

An external expert group was founded to analyze possible critical assessments and user safety. The group included one Omaolo developer nurse, 3 physicians with specialist degrees in general practice, 1 physician with a specialist degree in public health, 3 personnel working in the ESC development department, and 1 coder responsible for the Omaolo ESC production.

### Statistical Methods and Metrics for Assessing Triage Accuracy

Triage accuracy was calculated as the percentages of matches for each individual ESC and 95% CI. The calculations were based on the initial nurses’ triage assessments. The hypothesis is that the Omaolo ESC is safe to use [40].

We set the safe symptom assessment based on a previous study, as at most one level of urgency less urgent than the assessment of a nurse, and the performance of the ESC as 97% safe accuracy across all completed assessments [23]. We also assumed that misdiagnosis by physicians occurs in approximately 5% of outpatients [43]. We estimated the required sample size by assuming the given range of safe advice at 97% and using a 95% CI, and we computed the CI estimate for the true proportion of safe ESC assessments [23].

Triage comparison charts (confusion matrices) are created in which true positive (TP) represents the outcome where the model correctly predicts the positive class (condition is detected when present). True negative (TN) is the outcome where the model correctly predicts the negative class (condition is not detected when absent). False positive (FP) represents the outcome where the model incorrectly predicts the positive class (condition is detected when absent). False negative (FN) is the outcome where the model incorrectly predicts the negative class (condition is not detected when present). These values are crucial components in calculating the positive predictive values (PPVs), negative predictive values (NPVs), and Matthews correlation coefficient (MCC). The PPV, NPV, and MCC formulas are given as:

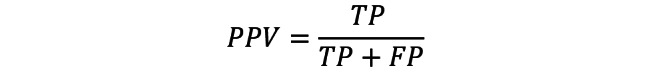

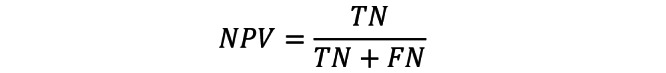

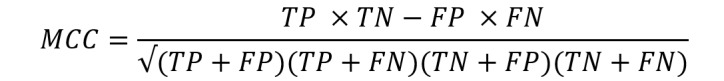







These values are typically extracted from a confusion matrix, as illustrated in Figure 3 and presented in Table 1 of the study.

**Table 1:** Results of matching the nurse triage (gold standard) with the Omaolo electronic symptom checker recommendation in 15 different symptoms^a^.

Absolute magnitude of the Matthews correlation coefficient	Interpretation
±0.00-0.10	Negligible correlation
±0.10-0.39	Weak correlation
±0.40-0.69	Moderate correlation
±0.70-0.89	Strong correlation
±0.90-1.00	Very strong correlation

^a^The Omaolo electronic symptom checker recommendation is defined as safe if the critical condition was not assessed as urgent or on-call duty by the nurse but assessed by the electronic symptom checker as nonurgent or self-care. The CI range in the column reporting the number of completed assessments is assumed at safe advice of 97% and using a 95% CI. The table also shows positive predictive values and negative predictive values following the Matthews correlation coefficient.

The MCC ranges from 1 to +1, where ±1 indicates perfect agreement or disagreement, and 0 indicates no relationship.

### Ethical Considerations

This study was reviewed by the Pirkanmaa hospital district’s ethics committee (ETL-Code: R18126), and regional permission was additionally granted by each participating organization, all according to the regulations of the University of Tampere.

The most significant ethical issue related to the research setting is that the user’s participation in the research does not affect their chances of receiving timely treatment. All users who fill out the symptom checker will be forwarded to an appointment with an experienced nurse. Denial of treatment for users who refused to participate in the study was strictly prohibited.

When users (patients) were recruited for this study, the research assistant informed the user about the study, distributed the study information sheet, and then asked if the user was willing to participate in the study. If, after being informed, the user was willing to participate in the study, they were asked to sign a consent form in which the user acknowledged that they had received sufficient information about the study and agreed to participate in it. The user was given an information sheet about the study with contact information in case the user wanted to ask more about the study. The user was paid no amount of compensation.

The patient user’s consent form was disconnected from the response form with a personal identification code, that is, the users were completely anonymized. No medical record data were collected or combined with research forms. The users could withdraw their consent to the study at any time. However, the completed forms cannot be destroyed after the data collection because the consent form containing the personal data does not have the identification code to identify the appropriate study forms to be destroyed.

## Results

### Overview

A total of 877 patient user assessment cases were successfully collected. No patient user identifying age or gender data were collected for this study.

### Quantifying and Comparing the Levels of Urgency and Triage Accuracy

The ESC’s and nurse’s triage were exactly matched in 53.7% (471/877; 95% CI 49.2%-55.9%) of the cases in all symptom assessments. Considering ESC’s individual main symptom triage suggestions, the most exactly matched assessments were found for shoulder pain, stiffness, or injury (32/47, 68%), dental or oral symptom or trauma (37/61, 61%), and sexually transmitted disease (23/39, 59%).

The mean value for exactly matched or overly conservative but suitable for all symptom assessments was 66.6% (584/877; 95% CI 63.4%-69.7%). Safe assessments of ESCs accounted for 97.6% (856/877; 95% CI 95.6%-98.0%) of all assessments made (Table 1, Figures 2-4). Concerning acute cases in which the nurse evaluated that a user needed to be treated urgently the ESC’s exactly matched accuracy was 70.9% (244/344; 95% CI 65.8%-75.7%), and in cases whether medical care should be sought or self-care is sufficient, matches were found in 65.9% of cases (351/533; 95% CI 61.7%-70.0%). Sensitivity for the Omaolo ESC was 62.6% and specificity was 69.2%. The proportions of evaluations occurred at a ratio of 100 suitable to 25 overtriage to 22 undertriage. The overly conservative triage (overtriage) suggestions made by the ESC occurred most often for respiratory tract infection in 44% (46/104), heartburn in 33% (8/24), and headache in 37% (15/41) of cases (Figure 4).

The question “Do you feel the need to change your assessment of the need for treatment (triage) after seeing the responses and recommendation from the ESC? (Yes/No)” was answered with the “Yes” option in 19 out of 877 assessments. In answering the follow-up question “If yes, why did you change the assessment of the need for treatment (triage) after seeing the responses and recommendation by the ESC?”, the nurses frequently stated that the symptoms they were told by the user did not match with the ones the user had stated while filling the ESC (Table 2 and Multimedia Appendix 2). In the last section, in the case of an affirmative answer, the path of changing the triage ended with “If you feel it is necessary to change your triage assessment, reselect where the user should be referred to according to the classification terms of the ESC recommendation.” In these 19 assessments, the nurses were found to have chosen a triage suggestion closer to that of the ESC assessment (Multimedia Appendix 2). In 80 cases across all completed 877 assessments, the triage nurse consulted a doctor in assessing the triage.

**Table 2:** Triage nurse form questions results, the y-axis represents the number of observations.

Symptom	Completed matched assessments, n	Percentage of safely matched advice symptom assessments, % (95% CI)	Exactly matched symptom assessments, % (95% CI)	Positive predictive value (precision), %	Negative predictive value, %	Matthews correlation coefficient
Anal region symptom	41	100 (91.4-100)	53.7 (37.4-69.3)	14.3	74.1	–0.133
Cough	71	95.8 (88.1-99.1)	50.7 (38.6-62.8)	52.2	48.1	0.004
Dental or oral symptoms or trauma	62	85 (74.2-93.1)	59.7 (46.5-72.0)	62.9	57.7	0.204
Diarrhea	21	95.2 (76.2-99.9)	45.5 (24.4-67.8)	0	66.7	–0.37
Discharge from the eye, watery or reddish eye	65	100 (90.0-99.6)	58.5 (45.6-70.6)	66.7	28.6	–0.036
Headache	41	100 (91.4-100)	53.7 (37.4-69.3)	50.0	66.7	0.138
Heartburn	24	100 (85.8-100)	37.5 (18.8-59.4)	26.7	55.6	–0.233
Knee symptom or injury	55	100 (93.5-100)	56.4 (42.3-69.7)	36.8	66.7	0.035
Lower back pain or injury	65	100 (94.5-100)	56.9 (44.0-69.2)	46.7	80.0	0.253
Painful or blocked ear	81	100 (95.6-100)	58.0 (46.5-68.9)	20.0	49.0	–0.115
Respiratory tract infection	104	94.2 (88.0-97.9)	41.3 (31.8-51.4)	40.2	45.4	–0.118
Sexually transmitted disease	39	100 (91.0-100)	59.0 (42.1-74.4)	50.0	61.3	0.093
Shoulder pain, stiffness, or injury	47	100 (92.5-100)	68.1 (52.9-80.9)	42.9	72.5	0.12
Sore throat or throat symptom	101	98 (93.0-99.8)	54.5 (42.3-62.5)	48.3	78.6	0.19
Urinary tract infection	60	100 (94.0-100)	51.7 (38.4-64.8)	57.1	46.9	0.04
In total	877	97.6 (96.4-98.5)	53.7 (50.3-57.1)	49.4	59.5	0.089

### The Analysis of Critical Assessments

In total, 21 critical assessments were identified for further analysis. Details are given in Multimedia Appendix 3. Further analysis showed that there were no indications that patient safety was endangered. In Figures 3 and 4, these patient user cases are marked as “Potentially unsafe” (resolved).

## Discussion

### Principal Findings

The findings suggest that while exact matches of the Omaolo ESC recommendations with the gold standard (nurse triage) occurred in just over half of the cases, nearly all cases were evaluated as safe, with urgency levels being at most 1 level less urgent compared with the nurses’ triage. Concerning acute assessments, an exact match was found in nearly 3 out of 4 cases. This study assessed for the first time the safety of the Omaolo ESC within the Finnish PHC context.

### Comparison to Previous Work

In a systematic review, the diagnostic accuracy and the triage-making abilities of ESC services such as Ada, Babylon, Buoy, K Health, Mediktor, Symptomate, and Your.MD were compared with those of general practitioners’ assessment using clinical case vignettes. The average safe operating recommendations ranged from 90.1% (SD 7.4%) [23]. By contrast, the general practitioner’s percentage of safe advice stood at 97.0% (SD 2.5%) [23]. Comparatively, the proportion of safe Omaolo ESC assessments across all investigated cases was 97.6% (856/877) using similar methods for safety assessment. These findings underscore the safety of Omaolo ESC compared with assessments by experienced nurses, particularly notable given the real-life setting of our study. By comparing Omaolo ESC’s accuracy to internationally reported results, we can gauge its overall performance and capabilities [8,22-24,44].

A study examining Ada’s use by 378 “walk-in” patients in urgent care compared its triage accuracy with that of a triage nurse using the Manchester Triage System, conducted under similar circumstances as the Omaolo ESC’s triage accuracy study. Ada exhibited an undertriage rate of 8.9% (34/378) and an overtriage rate of 57.1% (216/378). Out of 378 participants, 344 (91%) were triaged identically or more conservatively, while 34 (8.9%) were undertriaged by the app. The assessment was deemed safe in 94.7% (358/378) of patients when compared with the Manchester Triage System assessment. In the Omaolo ESC study involving 877 users, 726 (83%) were triaged identically or more conservatively, with 151 (17%) being undertriaged. Notably, Omaolo ESC exhibited a 29% (255/877) overtriage rate, with evaluations occurring at a ratio of 100 suitable evaluations to 25 overtriages to 22 undertriages. Compared with Ada, in the Omaolo study, overtriage rates were lower.

A recent systematic review concluded that the median accuracy of studied apps in determining the necessity of emergency care was 80% (IQR 74.6%-86.8%) [20]. For Omaolo ESC’s triage of acute cases where a nurse assessed urgent treatment as necessary, exact matches occurred in 244 out of 344 cases, representing 70.9% (95% CI 65.8%-75.7%) of cases, while matches indicating whether medical care should be sought or if self-care is sufficient occurred in 351 out of 533 cases, totaling 65.9% (95% CI 61.7%-70.0%) of cases. This is in line with the international figure of 73.3% (IQR 70.5%-82.3%) [8]. In addition, the median app sensitivity was 51.9% (IQR 40%-78.2%), and the median specificity was 93.3% (IQR 87.4%-96.4%) [8]. Omaolo ESC exhibited a sensitivity of 62.6% and a specificity of 69.2%.

ESC’s capability to detect individuals in need of immediate treatment is vital for user safety. In addition to that, an ESC that holds promise for safely assisting in self-triage and that helps prevent overcrowding of emergency departments could bring added value to health care. Notably, concerning Omaolo, the least overtriage was observed in chief symptoms of sexually transmitted diseases (4/39, 10.3%), shoulder pain stiffness or injury (4/47, 8.5%), and painful or blocked ears (8/81, 9.9%). Conversely, more sensitive and risk-averse chief symptom assessments such as headaches (16/41, 39%), heartburn (11/24, 45.8%), and respiratory tract infections (49/104, 47.1%) exhibited higher rates of false positives, raising concerns about overcrowding and possible unnecessary health care service use. However, due to potentially serious conditions, these ESCs are set to be sensitive in order to rule out alarming symptoms.

A relatively high PPV was found in cough, dental or oral symptoms or trauma, discharge from the eye or watery or reddish eye, and urinary tract infection assessments. This indicates reduced false positives, beneficial when false positives have high costs or the condition is not severe. High PPV minimizes overtreatment and unnecessary costs. Conversely, a moderate PPV found from other assessments is acceptable if follow-up tests are inexpensive and harmless precautionary measures are taken. For the symptom assessments of anal region symptoms, diarrhea, heartburn, headache, and sexually transmitted diseases a low number of cases has to be taken into consideration when evaluating these values.

A relatively high NPV was found in assessments of anal region symptoms, dental or oral symptoms or trauma, diarrhea, headache, heartburn, knee symptoms or injury, lower back pain or injury, sexually transmitted diseases, shoulder pain and stiffness or injury, and sore throat or symptom. High NPV is crucial for serious or contagious conditions needing early intervention, minimizing false negatives. Moderate NPV observed in this study for cough, discharge from the eye, watery or reddish eye, painful or blocked ear, respiratory tract infection, and urinary tract infection is acceptable if the condition is not severe or resolves on its own.

The MCC shows weak positive relationships for dental or oral symptoms or trauma and lower back pain or injury and weak negative relationships for heartburn and diarrhea. Other assessments have negligible relationships, suggesting the symptom checker’s predictions are slightly better than random guessing.

### Strengths and Limitations of the Study

The real-life setting presents both strengths and potential weaknesses for this study. There are notable concerns regarding potential selection bias. First, users completing the ESC in PHC center waiting rooms may experience different symptoms compared with those using the ESC outside of such environments. Moreover, while the Omaolo ESC is designed for users over the age of 15 years, only individuals over the legal age of 18 years were recruited for this study, potentially limiting the generalizability of findings. Furthermore, users who were unable to independently complete the ESC due to technological limitations (such as difficulty using a computer mouse or tablet devices) were excluded from the study, introducing another potential limitation.

In addition, the rarity of users with serious acute symptoms in this setting may skew the study results, as their symptoms may not be severe enough to interfere with ESC questionnaire completion. However, it is worth noting that the Omaolo ESC prompts users to urgently contact health care services if they are unable to complete the questionnaire due to the severity of their symptoms.

Furthermore, potential users with mild, self-treatable symptoms may have been excluded as such cases may remain unreported in this study context. Nonetheless, the study focused on completed ESC triage assessment accuracy, specificity, and sensitivity.

In some instances, the triage classification made by nurses may have been influenced by user-reported needs for sick leave certificates, potentially biasing the calculated ESC accuracy.

The data accumulation process was hindered by the COVID-19 pandemic, which slowed down research activities [45,46]. However, the exponential growth in individual Omaolo users during the pandemic, particularly with the use of the coronavirus ESC, was noted [47]. To address the scarcity of completed assessments for some ESCs and urgent cases, future research will supplement data with electronic patient cases, known as case vignettes. This approach will also allow for the assessment of ESC performance across rare symptoms and conditions in a primary care setting.

Finally, it is important to acknowledge that, for this study, the nurse’s triage assessment was considered the gold standard. An alternative approach could have been to use outcomes during follow-up, such as revisits or hospitalizations after 30 days, to determine the success of the triage.

### Implications and Future Research

In this study, heartburn and diarrhea were relatively infrequent as chief symptoms for safety and accuracy evaluations. However, it is crucial to monitor and supplement the safety assessments of these symptoms in future case vignette studies. Despite their limited representation in this study, the present results suggest that heartburn and diarrhea are likely safe to assess using the ESC. To address more common and less urgent situations, vignette studies will be instrumental. These vignettes, sourced from various contexts, will undergo thorough testing to ensure that individual vignettes’ difficulty and correlation with overall assessment are carefully considered [48].

Moving forward, there should be an evolution toward more standardized methodologies and studies tailored to specific settings. Regulation and standardization of evaluations are vital for ensuring the transferability of findings across different contexts [49]. In addition, adopting a patient-centered approach is essential for evaluating ESCs effectively. A standardized process with clear specifications for vignette-based clinical evaluation is necessary to guide developers and facilitate objective comparisons among ESCs. This approach will enhance the reliability and validity of ESC assessments and promote their widespread adoption in clinical practice [43,50].

The data suggests that the Omaolo ESC is reliable for preliminary symptom assessment and triage, demonstrating a high level of safety in aligning with triage recommendations from experienced nurses, especially in acute cases. Omaolo uses structured questions and fixed algorithms designed by professionals to provide medically qualified recommendations, though it does not use AI. The question of whether AI-based ESC systems would be a desirable advancement or introduce additional risk and uncertainty for patient safety is complex.

AI-based ESC systems have the potential to enhance efficiency and accuracy by continuously learning from vast datasets and adapting to evolving medical knowledge. They can rapidly process information and offer consistent assessments across different users and contexts, potentially covering a broader range of symptoms and conditions. However, there are risks associated with AI-based systems, including reliance on data quality and a potential lack of nuanced clinical judgment compared with human triage nurses.

While AI-based ESC systems can complement triage processes, human supervision, and oversight are essential to ensure patient safety. Human triage nurses provide contextual understanding, empathy, and critical thinking that AI systems may lack, intervening when AI-generated recommendations are uncertain or potentially harmful. Therefore, AI-based systems should be viewed as tools to augment rather than replace human triage nurses.

In conclusion, ESCs should augment traditional triage rather than substitute for it, potentially leading to benefits such as reduced phone calls and increased accessibility to health services. Omaolo ESC, with its acceptable specificity and accuracy, holds promise for preventing unnecessary use of primary health care services. In addition, well-structured ESC assessments systematically collect user medical history and symptom information, evidenced by triage nurses’ decisions to adjust triage based on additional user-provided information.

### Impacts of the Study on the Omaolo Electronic Symptom Checker Service

The results highlighting the safety of Omaolo have been crucial for the continuation of the ESC service in Finnish health care.

### Conclusions

The primary objectives of this study were to evaluate the safety and provide essential insights into the accuracy, specificity, and sensitivity of the Omaolo ESC. The results indicate that the ESC is safe for use compared with assessments conducted by triage nurses. Furthermore, the Omaolo ESC exhibits the potential to guide patients to appropriate triage destinations aptly, ensuring they receive timely and suitable care.

## Appendix

Multimedia Appendix 1 Quantitative clinical study.

Multimedia Appendix 2 Details of affirmative answers to “do you feel the need to change your assessment of need for treatment (triage) after seeing the responses and recommendation from the electronic symptom checker?".

Multimedia Appendix 3 Identified conflict situations (21 critical cases). The findings of the inspection, the identified conflict, the final result, and the declared final result (assessed by the evaluation team).

## Abbreviations

AI: artificial intelligence
ESC: electronic symptom checker
FIHW: Finnish Institute for Health and Welfare
FN: false negative
FP: false positive
GP: general practitioner
MSAH: Finnish Ministry of Social Affairs
NPV: negative predictive values
PHC: primary health care
PPV: positive predictive values
THL: Finnish Institute for Health and Welfare
TN: true negative
TP: true positive

## Data Availability

The electronic datasets generated during and analyzed in this study are available from the corresponding author upon reasonable request. Paper forms used to create these datasets are stored in a locked cabinet. The consent form containing the user’s name and signature is kept in a separate locked cabinet. All paper forms are stored in the Tampere University. The forms are disposed of with a shredder 10 years after the end of the study.
