# Supplementary material for: Triage Accuracy and the Safety of User-Initiated Symptom Assessment With an Electronic Symptom Checker in a Real-Life Setting: Instrument Validation Study
Source: JMIR Hum Factors. 2024 Sep 26;11:e55099. doi: 10.2196/55099 (PMC11467609; doi:10.2196/55099)
Supplement: Multimedia Appendix 1 [file humanfactors_v11i1e55099_app1.docx]

Appendix 1. Quantitative clinical study.

| **Differences between the nurse's and ESC’s (electronic symptom checker’s) triage in a real-life setting.** | | | |
| --- | --- | --- | --- |
| **Study type** | **Study objectives** | **Study design and setting** | **Outcome** |
| Quantitative validation study | Differences and similarities between the nurse's and ESC’s (N: 15) triage in real-life setting | Comparison of the nurse's and ESC’s triage (see Figure 2) | Triage frequencies, proportions, ESC safety, specificity, and sensitivity |
| **Research questions** | | | |
| Are the recommendations of the ESC safe to use (an assessment is defined as safe if conflict is not met: nurse’s triage is urgent or on-call duty but assessed by the ESC as non-urgent or self-care)? | | | |
| Accuracy: To what proportion (% and 95 % confidence intervals for the estimate) of the assessments do the experienced nurse's triage and the recommendation of action given by the ESC correspond or differ before the nurse has seen the recommendation of action given by the ESC? | | | |
| Did an experienced nurse change his or her assessment after seeing the recommendation for action from the ESC? | | | |
| What are the key factors that lead an experienced nurse’s triage to differ from the ESC recommendation? | | | |
| Undertriage: How many assessments are there and to which ESC do they relate where a triage nurse would refer a user to a heavier service than the ESC recommends (two levels lighter is considered as an unsafe recommendation by the ESC)? | | | |
| Overtriage: How many assessments are there and to which ESC do they relate where the triage nurse would refer the user to a lighter service than the ESC recommends (two levels heavier is considered as an overly conservative recommendation for an ESC)? | | | |
| Analysis of the content for the reasons given by the nurses that influenced their triage when the individual assessments differed. | | | |
