# Supplementary material for: Triage Accuracy and the Safety of User-Initiated Symptom Assessment With an Electronic Symptom Checker in a Real-Life Setting: Instrument Validation Study
Source: JMIR Hum Factors. 2024 Sep 26;11:e55099. doi: 10.2196/55099 (PMC11467609; doi:10.2196/55099)
Supplement: Multimedia Appendix 2 [file humanfactors_v11i1e55099_app2.docx]

Multimedia Appendix 2. Details of affirmative answers to “do you feel the need to change your assessment of need for treatment (triage) after seeing the responses and recommendation from the electronic symptom checker?”

Nurse could choose: Self-care (with instructions); Book an appointment with a healthcare professional (non-urgent); Will be treated at this center, non-urgent; Will be treated at this center, urgent; Go to the emergency room, urgent. Same options applied in reselecting where the user should be referred to according to the classification terms of the electronic symptom checker’s recommendation. In the last column the ESC’s triage is revealed.

| **Choose where the patient should be directed (nurse’s triage):** | **If yes, why did you change the assessment of the need for treatment (nurse’s triage) after seeing the responses and recommendation from the electronic symptom checker?** | **If you feel it is necessary to change your triage assessment, reselect where the user should be referred to according to the classification terms of the electronic symptom checker’s recommendation** | **Symptom checker's recommendation of action** |
| --- | --- | --- | --- |
| Will be treated at this center, non-urgent | If I had only seen the symptom checker evaluation, I would have directed the triage to self-care. There were no indications of sinusitis, which was eventually treated. | Will be treated at this center, non-urgent | P4. May be referred to a doctor during office hours [72h] |
| Self-care (with instructions) | The patient didn't mention some symptoms, but these were included in the symptom assessment. | Will be treated at this center, urgent | P0. Treated immediately, contact emergency center [0h] |
| Book an appointment with a healthcare professional (non-urgent) | The patient mentioned swelling (symptom of infection) and has pain. | Will be treated at this center, urgent | P2. Treated at an emergency center, but not during night [10h] |
| Will be treated at this center, non-urgent | The patient forgot to mention the sore throat symptom, but this was mentioned in the symptom assessment. | Will be treated at this center, urgent | P1. Contact emergency center services [2h] |
| Self-care (with instructions) | In the symptom checker, long-term symptoms are described as worse than when asked during the appointment. According to the information on the symptom checker form, I would make an immediate appointment. | Book an appointment with a healthcare professional (non-urgent) | P2. Treated at an emergency center, but not during night [10h] |
| Self-care (with instructions) | Symptoms are described as worse and more severe than when asked during the appointment. | Will be treated at this center, non-urgent | P3. May be referred to a doctor during office hours [24h] |
| Will be treated at this center, non-urgent | Different symptoms in the symptom checker. | Go to the emergency room, urgent | P0. Treated immediately, contact emergency center [0h] |
| Will be treated at this center, non-urgent | Overall worsened state. Not able to pin chin to the chest and fever. | Will be treated at this center, urgent | P0. Treated immediately, contact emergency center [0h] |
| Will be treated at this center, non-urgent | In the symptom checker questions, he described a sudden onset of severe pain and visual disturbance, which were not mentioned in the appointment. | Will be treated at this center, urgent | P0. Treated immediately, contact emergency center [0h] |
| Go to the emergency room, urgent | Feet problem, 2 days of non-emergency appointment. | Book an appointment with a healthcare professional (non-urgent) | P4. May be referred to a doctor during office hours [72h] |
| Will be treated at this center, non-urgent | The pain is more detailed in the electronic symptom assessment than was found in the appointment. | Will be treated at this center, urgent | P2. Treated at an emergency center, but not during night [10h] |
| Book an appointment with a healthcare professional (non-urgent) | Different symptoms in the symptom checker. | Will be treated at this center, urgent | P2. Treated at an emergency center, but not during night [10h] |
| Self-care (with instructions) | CRP and fever. It is found out that the symptoms are caused by a viral respiratory infection. | Book an appointment with a healthcare professional (non-urgent) | P0. Treated immediately, contact emergency center [0h] |
| Self-care (with instructions) | Blocked ear caused by wax accumulation. Flu non-related (started two days ago). | Self-care (with instructions) | L2-L4. Start self-care (with instructions) |
| Go to the emergency room, urgent | Severe pain for which pain medication periodically helps. | Will be treated at this center, urgent | P3. May be referred to a doctor during office hours [24h] |
| Self-care (with instructions) | General flu is sufficiently self-treatable, but patient needs a sick leave certification. | Will be treated at this center, urgent | P0. Treated immediately, contact emergency center [0h] |
| Self-care (with instructions) | Ear wax accumulation can be self-treated with self-remedy equipment. No signs of infection. | Will be treated at this center, non-urgent | P3. May be referred to a doctor during office hours [24h] |
| Go to the emergency room, urgent | No explanation. | Book an appointment with a healthcare professional (non-urgent) | P3. May be referred to a doctor during office hours [24h] |
| Will be treated at this center, non-urgent | Patient hasn't been practicing sufficient self-care, which would otherwise suffice. Wants an appointment. | Self-care (with instructions) | L2-L4. Start self-care (with instructions) |
