# Supplementary material for: Triage Accuracy and the Safety of User-Initiated Symptom Assessment With an Electronic Symptom Checker in a Real-Life Setting: Instrument Validation Study
Source: JMIR Hum Factors. 2024 Sep 26;11:e55099. doi: 10.2196/55099 (PMC11467609; doi:10.2196/55099)
Supplement: Multimedia Appendix 3 [file humanfactors_v11i1e55099_app3.docx]

Multimedia Appendix 3. Identified conflict situations (21 critical cases). The findings of the inspection, the identified conflict, the final result, and the declared final result (assessed by the evaluation team).

| **Description of the critical case** | **Conflict** | **Evaluation and analysis** |
| --- | --- | --- |
| **Respiratory tract infection:** Prolongation of flu symptoms is the nurse's most significant reason for making an observation that deviates from the symptom assessment. However, the form states that the duration of the symptoms is 4 days. The form states no critical symptoms. | Assessed by the nurse to be treated urgently, while the result of the symptom checker was interpreted as self-treatment: "Your symptoms may be caused by a sinus infection. See Health library's article on Sinusitis. If 7-10 days after the start of the flu your symptoms get worse, you feel pain in the deep part of your face or if you have a pounding cough especially at night, the nose is blocked, the discharge is colored or the sense of smell has deteriorated, you should see a professional for an echocardiogram of the sinuses. Rhinitis caused by a virus is always accompanied by inflammation of the nasal sinuses and the accumulation of mucus in the sinuses in the early days. That's why an echocardiogram should not be done in the early stages of symptoms, but only if healing does not progress normally. Sometimes an inflammation in the area of ​​the root of an inflamed tooth may spread into the sinus. Dental sinusitis can occur without a runny nose or other symptoms of respiratory infections. However, it is usually accompanied by sensitivity in the tooth. You can get more information about hoarseness and its self-care in the Health Library's article "Hoarseness or loss of voice." | It is found that the symptom checker in this particular case works clinically as it should when the information given on the form is correctly reported. |
| **Respiratory tract infection:** Overall condition. Quality and duration of symptoms. This background information was the nurse's most significant reason for making an observation that deviates from the symptom assessment. The form states that the duration of the symptoms is 14 days. The form states no critical symptoms. |  | There are no indications that patient safety was endangered. The user filled 14 days as the duration of the symptoms. If so, the interpretation logic guides the user to contact a professional. |
| **Respiratory tract infection:** The form states that the duration of the symptoms is 10 days. The form states no critical symptoms. |  | There are no indications that patient safety was endangered. The user filled 10 days as the duration of the symptoms. If so, the interpretation logic guides the user to contact a professional. |
| **Respiratory tract infection:** The form states that the duration of the symptoms is 9 days. The form states no critical symptoms. |  | There are no indications that patient safety was endangered. The user filled 9 days as the duration of the symptoms. If so, the interpretation logic guides the user to contact a professional. |
| **Respiratory tract infection:** The form states that the duration of the symptoms is 7 days. The form states no critical symptoms. |  | There are no indications that patient safety was endangered. The user filled 7 days as the duration of the symptoms. If so, the interpretation logic guides the user to contact a professional. |
| **Respiratory tract infection:** Nurse reported an ear infection finding. It was difficult for the patient to choose a suitable symptom assessment, but they found the right one in the end. The symptom assessment suggested self-treatment. |  | According to the nurse, the patient's ear had an infection. Otitis media often heals on its own. If there are significant symptoms, the symptom directs the user to contact a professional. There are no indications that patient safety has been compromised. |
| **Sore throat or throat symptom:** The nurse assessed it as urgent; the result of the symptom checker was interpreted as self-treatment. | The feedback reads: If your symptoms persist or worsen, you can contact a professional evaluation in the next few days or, if necessary, sooner. | There are no indications that patient safety was threatened. It is stated that the symptom checker in this clinical triage assessment case works as it should. |
| **Sore throat or throat symptom:** The nurse assessed it as urgent; the result of the symptom checker was interpreted as self-treatment. | The nurse's most significant reason for making an observation that deviates from the symptom checker: the patient's need for a sick leave medical certificate. | The need for sick leave could be asked separately in all Omaolo symptom checkers. However, this does not affect the triage. There are no indications that patient safety was threatened in this case. |
| **Cough:** The nurse assessed it as urgent; the result of the symptom checker was interpreted as self-treatment sufficient. | If your symptoms get worse or last longer or new symptoms appear, fill out the symptom checker again if necessary or contact a healthcare professional. | The instructions given by the symptom checker are sufficient. The if clause of the feedback gives instructions in such a situation. There are no indications that patient safety was threatened. |
| **Cough**: The nurse assessed it as urgent; the result of the symptom checker was interpreted as self-treatment sufficient. | You have a cough and also heartburn (acid rising in the throat). Read more about the articles in the Health Library: Heartburn and Cough. Has your heartburn been treated with the methods in the article? You can also fill out the heartburn symptom assessment to get a more accurate assessment of the need for treatment. If your symptoms get worse or last longer or new symptoms appear, fill out the symptom assessment again if necessary or contact a healthcare professional. | The result of the symptom checker also falls into category P3 or P4 (Not urgent). There are no indications that patient safety has been compromised. |
| **Cough:** Assessed by the nurse as urgent; the symptom checker interpreted as non-urgent (treated non-urgently). | The most significant reason/reasons reported by the nurse in their assessment: shortness of breath, prolongation of symptoms, smoking. | The symptom checker of clinical triage works as it should when the information given on the form is correct. The symptoms reported by the user on the symptom checker form are significantly different from the symptoms described by the nurse. |
| **Diarrhea:** The most significant reason reported by the nurse: Duration of symptoms. The nurse assessed it as urgent, the symptom checker as self-treatment. | The result of the symptom checker: "You probably have a virus-caused, self-limiting illness. The incubation period of the disease is short, usually from 12 hours to two days. Rotavirus and norovirus cause a very similar disease. The disease usually differs from bacterial diarrhea in that the symptoms of viral diarrhea begin very suddenly, and in addition to diarrhea, almost everyone has nausea or vomiting, and often also fever or a feverish feeling with aches. Follow good hand hygiene and read the article on self-treatment of diarrhea in the Health Library. Contact a professional if the stomach pain is severe, there is bloody diarrhea, or the diarrhea is accompanied by other symptoms such as nausea or a slight temperature.” | No indication that patient safety has been compromised (no alarming symptoms). |
| **Dental or oral symptom or trauma:** Implied and subjective pain symptoms are the nurse's most significant reasons for making an observation that deviates from the symptom assessment. However, the form states no findings of pain. The form states no critical symptoms. | Assessed by the dental nurse to be treated urgently, the result of the symptom checker was interpreted as non-urgent: "Based on the symptom assessment, you have been diagnosed with a problem that requires evaluation by an oral healthcare professional within a month." | There are no indications that patient safety was endangered. However, the form is missing a VAS scale. |
| **Dental or oral symptom or trauma:** Overall condition. Quality and duration of symptoms. Acute and severe pain. Swelling. Pain medication helps for a short period of time. This background information was the nurse's most significant reason for making an observation that deviates from the symptom assessment**.** | Assessed by the dental nurse to be treated urgently, the result of the symptom checker was interpreted as non-urgent: "Based on the symptom assessment, you have been diagnosed with a problem that requires an evaluation by an oral healthcare professional within the next day." | There are no indications that patient safety was endangered. However, the form is missing a VAS scale. |
| **Dental or oral symptom or trauma:** Toothache, bite- and palpation sensitivity. Upper jaw pain, no need for pain medication. Gums sore and swollen. |  | There are no indications that patient safety was endangered. However, the form is missing a VAS scale. |
| **Dental or oral symptom or trauma:** Dental abscess next to a tooth. User has selected "there's a painless lump inside my mouth." The form shows no critical symptoms. |  | There are no indications that patient safety was endangered. The user reported the finding as an undefined lump. Thus, the interpretation logic guides the user to contact a professional. |
| **Dental or oral symptom or trauma:** Fluctuating pain. The form shows no critical symptoms. | Assessed by the dentist to be treated urgently, the result of the symptom checker was interpreted as non-urgent: “Based on the symptom assessment, you have been diagnosed with a problem that requires an evaluation by an oral healthcare professional within a month.” | There are no indications that patient safety was endangered. However, the form is missing a VAS scale. |
| **Dental or oral symptom or trauma:** Pain in lower jaw teeth, radiating pain, hot and cold sensitivity, caries finding in upper jaw teeth, gingivitis, gum bleeding, patient has taken pain medication, which helps with symptoms. On the form, the user has selected a single symptom as compared to the nurse’s assessment of several symptoms. | Assessed by the nurse to be treated urgently, the result of the symptom checker was interpreted as non-urgent: “Based on the symptom assessment, you have been diagnosed with a problem that requires an evaluation by an oral healthcare professional within a month.” | User has selected a single symptom, even though it is specified that several can be chosen. If there are significant symptoms, the symptom directs the user to contact a professional. There are no indications that patient safety has been compromised. |
| **Dental or oral symptom or trauma:** Toothache / pain in the upper jaw teeth. User had trouble choosing. User comments: My coming to the emergency room is about a longer-term problem that has been treated but is still ongoing. So I have received a partial treatment recommendation on the phone before and from the doctor. | Assessed by the nurse to be treated urgently, the result of the symptom checker was interpreted as non-urgent: “Based on the symptom assessment, you have been diagnosed with a problem that requires an evaluation by an oral healthcare professional within a month.” | Currently, the symptom checker does not take into account if it is an old symptom that still persists or has been partially treated. The form is also missing a VAS scale. |
| **Dental or oral symptom or trauma:** Upper jaw wisdom tooth ache. User implied: Unnecessary and pointless descriptions of symptoms. | Assessed by the dentist to be treated urgently, the result of the symptom checker was interpreted as non-urgent: "Based on the symptom assessment, you have been diagnosed with a problem that requires an evaluation by an oral healthcare professional within a month." | There are no indications that patient safety was threatened in this case. |
| **Dental or oral symptom or trauma:** Pain in upper jaw tooth, hot and cold sensitivity, toothache while brushing, no need for pain medication. User also mentions swelling sensation in cheek. | Assessed by the dentist to be treated urgently, the result of the symptom checker was interpreted as non-urgent: "Based on the symptom assessment, you have been diagnosed with a problem that requires an evaluation by an oral healthcare professional within a month." | User has selected a single symptom, even though it is specified that several can be chosen. If there are significant symptoms, the symptom directs the user to contact a professional. There are no indications that patient safety has been compromised. |
